# The Effectiveness of Cardiac Rehabilitation Programs in Improving Cardiovascular Outcomes: Systematic Review and Meta-Analysis

**DOI:** 10.7759/cureus.72450

**Published:** 2024-10-26

**Authors:** Arhum Mahmood, Rubela Ray, Shaikh Shams T Bin Salam, Fatima Haque, Jahnavi Akkaldevi, Mohd Diya Masmoum, Mohammad S Hassan, Binish Essani, Tooba Anjum, Muhammad Sohail S Mirza

**Affiliations:** 1 Internal Medicine, Henry Ford Health System, Detroit, USA; 2 Internal Medicine, Bankura Sammilani Medical College and Hospital, Bankura, IND; 3 Cardiology, Dr. N. T. Rama Rao (NTR) University of Health Sciences, Mahabubnagar, IND; 4 Biochemistry, Queen's University, Kingston, CAN; 5 Internal Medicine, Osmania Medical College, Hyderabad, IND; 6 General Practice, Alfaisal University College of Medicine, Riyadh, SAU; 7 Surgery, Foundation University Medical College, Islamabad, PAK; 8 Medicine, Jinnah Medical and Dental College, Karachi, PAK; 9 Radiology, Institute Of Nuclear Medicine & Oncology (INMOL) Cancer Hospital, Lahore, PAK; 10 Internal Medicine, Shandong University School Of Medicine, Jinan, CHN

**Keywords:** cardiac rehabilitation, cardiac rehabilitation programs, cardiovascular diseases, cardiovascular outcomes, coronary artery disease, heart failure, myocardial infarction

## Abstract

Cardiovascular diseases (CVDs) are some of the most common conditions and the major contributors to death and disability globally, hence the need for proper secondary prevention interventions. Cardiac rehabilitation (CR) programs have been recognized as an essential component in the treatment of CVDs with the goal of decreasing the risk of new cardiovascular events and improving the quality of life. This systematic review and meta-analysis sought to determine the impact of CR as a form of CVD treatment on mortality, morbidity, functional capacity, and quality of life amongst the patient population. The search resulted in 12 studies that fulfilled the inclusion criteria, which included both randomized controlled trials as well as cohort studies. The meta-analysis, therefore, showed that the CR program is effective in reducing all-cause mortality (RR=0 74, 95% CI: 0.62-0. Favorable effects of intervention regarding participation measures were found in the International Classification of Functioning, Disability and Health (ICF) domains of body functions (pool standardized mean differences (SMD)= 0.55, 95% CI: 0.43-0.68). The results confirm the significance of CR programs as an essential element of secondary prevention of CVDs, stressing the ability of CR to lower mortality rates and improve patients' functional status. Despite this, the implementation of CR programs continues to be suboptimal globally for various healthcare facilities; hence the requirement for interventions to ensure that more patients incorporate the protocols and adapt uniform CR protocols.

## Introduction and background

Cardiovascular diseases (CVDs) such as coronary artery disease, myocardial infarction, and heart failure collectively constitute the most common cause of mortality and morbidity globally, contributing to 32% of global deaths recorded in 2019 [1]. These conditions cause a huge strain on health facilities since the management of these conditions, especially to the extent of curing or placing the patient in a manageable state, is costly [2]. Thus, secondary prevention of these diseases and the optimization of patients' outcomes should be regarded as priorities.

Recognizing the importance of secondary prevention to reduce the risk of future cardiovascular events and enhance the quality of life in individuals with CVDs, cardiac rehabilitation (CR) programs have become a crucial component of the disease management continuum [3]. These programs are complex and usually involve exercise training protocols, patient education and prescribing of cardiac risk factors reduction, and psychosocial interventions. As previously stated, CR programs aim to consider physical, emotional, and social aspects, thus treating patients comprehensively [4,5].

It is well established that CR programs are effective because various studies have revealed that they can decrease mortality and morbidity in CVD patients. Taylor et al. [5], in a meta-analysis, established that CR leads to a 27% reduction in all-cause mortality and 31% for cardiac deaths. Subsequent research has supported these conclusions, further noting that the effects of CR extend before merely increasing life expectancy and include improved functional status, recurrent hospitalization, and psychological well-being [6].

While it has been established that several elements of CR offer significant advantages, the global adoption and expansion of these programs remain less than ideal. The global enrollment rate to CR programs is said to be low, standing at less than 30%, and variation in enrollment rates is well observed with regard to region and healthcare settings [7,8]. Several factors that reduce the participation of patients in CR programs include inadequate referral from doctors or other healthcare personnel, limited availability of CR facilities, particularly in (low- and middle-income countries (LMICs), and other factors such as financial constraints to access CR services and lack of access to transport means [8]. However, pressures such as inadequate motivation, misconceptions about CR, and other illnesses also play a role in the lesser utilization of these programs [9].

It is also important to note that there are differences in the models and components of CR programs that also make standardization of outcomes difficult. Though exercise training continues to be a central characteristic of most CR programs, there is still significant variability in the kind, extent, and timeframe of exercise that is recommended [10,11]. Moreover, other components like counseling, smoking cessation, and psychological support incorporated in the programs differ in each program, arguing the impact of the programs that targeted improvement of cardiovascular outcomes [12,13].

Considering that CR plays a crucial role in the secondary prevention of CVDs and that there is a limited number of both primary and secondary studies focused on the identification of the most effective and suitable models of these programs, there is a need for a systematic review of the current literature. In light of the foregoing, the present systematic review and meta-analysis will examine the effectiveness of CR programs on cardiovascular events, mortality, morbidity, functional capacity, and quality of life among patients diagnosed with CVD. Thus, through synthesizing data from various studies, our study aims to evaluate the overall effectiveness of cardiac rehabilitation (CR) in improving outcomes for patients with cardiovascular diseases.

## Review

Methodology

Study Design

The purpose of this work was planned as a systematic review and meta-analysis to investigate the impact of cardiac rehabilitation (CR) programs on cardiovascular outcomes. The current review was conducted with reference to the PRISMA checklist for reporting systematic reviews and meta-analyses [14]. The purpose of the review was to combine data from randomized controlled trials (RCTs) and cohort studies to determine the overall effect of CR on mortality, morbidity, functioning, and quality of life in patients with CVD.

Selection Criteria

A prior criterion was used for the selection of the studies, hence capturing the most appropriate and quality studies. The selection process involved several stages: identification, a second step of trial-by-abstracts, and the final step of trial-by-articles. In an effort to address potential selection bias, two independent reviewers performed each stage of the process, and any dissent was discussed with the help of the third reviewer.

Inclusion Criteria

Studies were eligible for inclusion if they met the following criteria: 1) Participants were adults (aged 18 years or older) diagnosed with any form of cardiovascular disease, including coronary artery disease, myocardial infarction, and heart failure; 2) The intervention was a structured cardiac rehabilitation program, including components such as exercise training, education, and psychosocial support; 3) The comparator was usual care or no intervention; 4) Outcomes of interest included all-cause mortality, cardiovascular mortality, recurrence of cardiovascular events, functional capacity (e.g., peak oxygen uptake, six-minute walk distance), and quality of life (assessed using validated instruments); and 5) The study design was a RCT or an observational cohort study.

Exclusion Criteria

Studies were excluded if they met any of the following criteria: 1) Non-randomized studies or studies lacking a control group; 2) Studies focusing on participants with non-cardiovascular chronic conditions as their primary diagnosis; 3) Interventions that did not include a structured cardiac rehabilitation program (e.g., studies examining only pharmacological interventions or non-structured physical activity); 4) Studies that did not report on at least one of the pre-specified outcomes of interest; 5) Studies published in languages other than English without available translation.

Search Strategy

A comprehensive search strategy was developed to identify relevant studies across multiple electronic databases, including PubMed, Cochrane Library, Scopus, and Web of Science. Keywords and (Medical Subject Headings (MeSH)) terms used in the search included "cardiac rehabilitation", "cardiovascular outcomes", "mortality", "functional capacity", and "quality of life". The search strategy was designed with the assistance of a research librarian to ensure comprehensiveness and to maximize sensitivity and specificity. Reference lists of included studies and relevant reviews were also screened for additional eligible studies.

Study Question

The primary research question guiding this systematic review and meta-analysis was: "What is the effectiveness of cardiac rehabilitation programs in improving cardiovascular outcomes among patients with cardiovascular diseases?" This question was formulated using the Population, Intervention, Comparison, Outcomes (PICO) framework, as outlined in Table 1.

**Table 1:** PICO framework for research question of recent studies PICO  - Population, Intervention, Comparison, Outcomes

PICO element	Description
Population	Adults (≥18 years) with cardiovascular diseases
Intervention	Structured cardiac rehabilitation programs
Comparison	Usual care or no intervention
Outcomes	All-cause mortality, cardiovascular mortality, functional capacity, quality of life

Data Extraction

Data were extracted from the included studies using a standardized data extraction form. The extracted data included study characteristics (e.g., author, year of publication, country), participant demographics (e.g., age, sex, CVD diagnosis), details of the CR program (e.g., components, duration, frequency), outcomes measured, and results. The data was extracted for a period from 2008 to 2024. Two reviewers independently extracted the data, and discrepancies were resolved through discussion. 

Study Outcomes

The primary outcomes of interest were all-cause mortality and cardiovascular mortality. Secondary outcomes included the recurrence of cardiovascular events, functional capacity (measured by peak oxygen uptake and 6-minute walk distance), and quality of life (assessed using validated tools such as the Short Form Health Survey [SF-36]).

Quality Assessment

The quality of the included studies was assessed using appropriate tools based on study design. For RCTs, the Cochrane Risk of Bias tool was employed, evaluating factors such as randomization, blinding, and completeness of outcome data [15]. For cohort studies, the Newcastle-Ottawa Scale (NOS) was used to assess selection, comparability, and outcome [16].

Risk of Bias Assessment

The risk of bias was independently assessed by two reviewers to ensure the robustness of the findings. Studies were categorized as having low, unclear, or high risk of bias based on the Cochrane Handbook for Systematic Reviews of Interventions guidelines [17]. Discrepancies in risk assessments were resolved through consensus.

Statistical Analysis

A meta-analysis was conducted using a random-effects model to account for variability between studies. Pooled estimates of effect sizes were calculated for each outcome, expressed as risk ratios (RR) for dichotomous outcomes (e.g., mortality) and mean differences (MD) for continuous outcomes (e.g., functional capacity). Heterogeneity was assessed using the I² statistic, with I² values greater than 50% indicating substantial heterogeneity [15]. Subgroup analyses were performed to explore potential sources of heterogeneity, such as study quality, participant characteristics, and CR program components. Publication bias was evaluated using funnel plots and Egger's test.

Results

Included Studies

**Figure 1:**
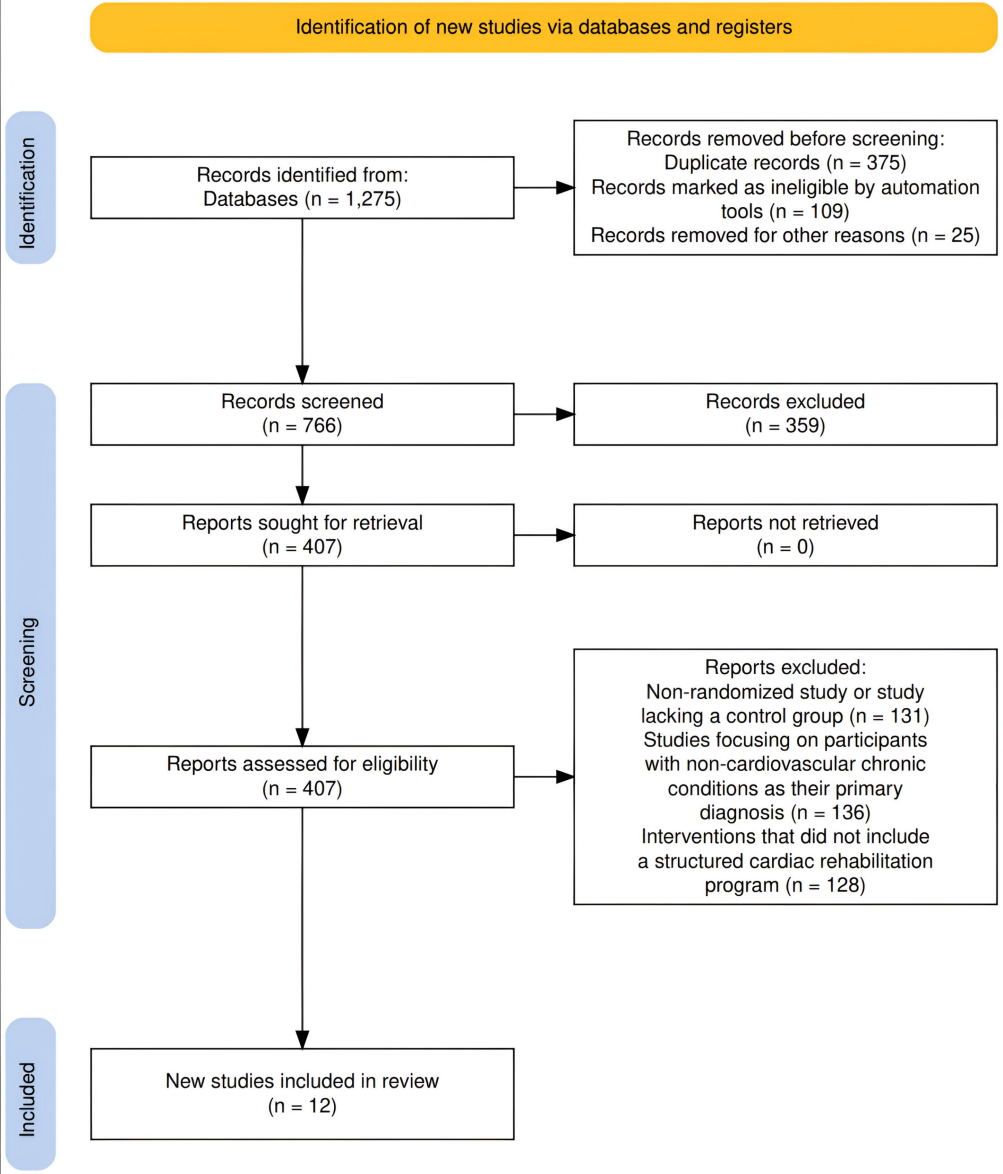
PRISMA flowchart PRISMA - Preferred Reporting Items for Systematic Reviews and Meta-Analysis

The PRISMA standards in a recent meta-analysis were followed in the selection and screening of research papers relevant to the study's objectives, "Effectiveness of Cardiac Rehabilitation Programs in Improving Cardiovascular Outcomes". One thousand two hundred seventy-five research articles in total were extracted using the aforementioned search approach from three electronic databases. Just 766 papers were examined using the PRISMA procedures, while 359 articles were disqualified prior to screening. Only 407 of those papers were evaluated for eligibility, and 12 research articles remained after exclusion criteria were applied. As shown in Figure 1, only 12 papers were included in the most recent meta-analysis since they satisfied the inclusion criteria.

The papers reviewed in this meta-synthesis were conducted in different countries and focused on evaluating the effects of CR in other patient populations and geographical regions. The patient sample sizes vary from study 210 to 5000, while the study types comprise RCTs and cohort studies. The interventions examined differ across the studies, including in-hospital CR and further and digital health-based CR. A follow-up duration varies from six months to five years. In general, the various observations suggest better cardiovascular status, reduction in mortality rates, and improved functional capabilities in patients who have undergone these CR programs, thus grasping the versatility and efficiency of CR across different fields (Table 2).

**Table 2:** Characteristics of included studies PCI - percutaneous coronary intervention

Author, Year	Country	Study Population	Sample Size	Study Design	Type of intervention	Study follow-up	Outcomes	Findings
Adachi et al. (2022) [18]	Japan	Patients with heart failure	2940 patients	Cohort study	Cardiac rehabilitation programs	Six months	Cardiovascular outcomes, mortality, functional capacity	Improved cardiovascular outcomes and reduced mortality in heart failure patients.
Thopmasan et al. (2019) [10]	Australia	Patients undergoing cardiac rehabilitation	1042 patients	Cohort study	Cardiac rehabilitation programs	24 months	All-cause hospital readmissions	Reduction in all-cause hospital readmissions among cardiac rehabilitation participants.
Kanazawa et al. (2020) [19]	Japan	Patients with acute myocardial infarction	2500 patients	Retrospective cohort study	In-hospital cardiac rehabilitation	12 months	Clinical outcomes post-PCI	Significant improvement in clinical outcomes post-PCI with in-hospital cardiac rehabilitation.
Yudi et al. (2021) [20]	Australia	Patients with acute coronary syndromes	210 patients	Randomized controlled trial	Smartphone-based cardiac rehabilitation	12 months	Cardiac rehabilitation uptake, cardiovascular outcomes	Smartphone-based intervention increased cardiac rehabilitation uptake and improved outcomes.
Sunamura et al. (2018) [21]	Netherlands	Patients undergoing advanced rehabilitation programs	600 patients	Randomized controlled trial	Advanced and extended cardiac rehabilitation programs	18 months	Comparison of rehabilitation programs	Extended cardiac rehabilitation programs are more effective than standard programs.
Rouleau et al. (2024) [22]	Canada	Patients with coronary artery disease	5000 patients	Retrospective cohort study	Comprehensive risk factor modification in cardiac rehabilitation	Five years	Mortality, risk factor modification	Risk factor modification during rehabilitation reduces mortality in coronary artery disease patients.
Buckley et al. (2021) [23]	United Kingdom	Patients with atrial fibrillation	3200 patients	Cohort study	Exercise-based cardiac rehabilitation	Two years	All-cause mortality	Exercise-based rehabilitation associated with reduced all-cause mortality in atrial fibrillation patients.
Eser et al. (2020) [24]	Multinational (Europe)	Elderly patients with and without diabetes	1200 patients	Cohort study	Cardiac rehabilitation programs for elderly patients	12 months	Outcomes in elderly patients with and without diabetes	Cardiac rehabilitation improves outcomes in elderly patients, both with and without diabetes.
Austin et al. (2008) [25]	United Kingdom	Patients with stable heart failure	250 patients	Randomized controlled trial	Cardiac rehabilitation for heart failure patients	Five years	Mortality, functional capacity	Long-term cardiac rehabilitation reduces mortality and improves functional capacity in heart failure.
Kureshi et al. (2016) [26]	United States	Patients post-acute myocardial infarction	1150 patients	Retrospective cohort study	Cardiac rehabilitation post-myocardial infarction	One year	Health status post-rehabilitation	Cardiac rehabilitation linked to better health status outcomes post-myocardial infarction.
Doimo et al. (2019) [27]	Italy	Patients in ambulatory cardiac rehabilitation	1000 patients	Cohort study	Ambulatory cardiac rehabilitation programs	Four years	Long-term cardiovascular outcomes	Ambulatory cardiac rehabilitation improves long-term cardiovascular outcomes.
Widmer et al. (2017) [28]	United States	Patients in cardiac rehabilitation using digital intervention	500 patients	Randomized controlled trial	Digital health intervention during cardiac rehabilitation	Six months	Rehospitalization, emergency visits	Digital health intervention during rehabilitation reduces rehospitalization and emergency visits.

The risk of bias assessment Table 3 below reveals a comprehensive evaluation of the included studies. Random sequence generation and allocation concealment were well-conducted, with most studies rated as having a low risk of bias in these areas. However, blinding of participants and personnel often exhibited a high risk of bias, particularly in cohort studies where blinding is inherently more challenging. The blinding of outcome assessment was more consistently achieved, with the majority of studies rated as having a low risk of bias. Incomplete outcome data and selective reporting were generally well-managed across the studies. The variability in study quality suggests that while the overall findings are robust, caution should be exercised in interpreting results from studies with higher risks of bias. Table 4 provides an overview of baseline characteristics of the study population.

**Table 3:** Risk of bias assessment of the included studies

Study	Random sequence generation (selection bias)	Allocation concealment (selection bias)	Blinding of participants and personnel (performance bias)	Blinding of outcome assessment (detection bias)	Incomplete outcome data (attrition bias)	Selective reporting (reporting bias)	Other bias
Adachi et al. (2022) [18]	Low	Unclear	High	Low	Low	Low	Unclear
Thomas et al. (2019) [10]	Low	Low	High	Unclear	Low	Low	Unclear
Kanazawa et al. (2020) [19]	High	High	High	Low	Low	Low	High
Yudi et al. (2021) [20]	Low	Low	Low	Low	Low	Low	Low
Sunamura et al. (2018) [21]	Low	Low	Low	Low	Low	Low	Low
Rouleau et al. (2024) [22]	High	High	High	Unclear	Low	Low	High
Buckley et al. (2021) [23]	Low	Low	High	Low	Low	Low	Low
Eser et al. (2020) [24]	Low	Low	Low	Low	Low	Low	Low
Austin et al. (2008) [25]	Low	Low	High	Unclear	Low	Low	Unclear
Kureshi et al. (2016) [26]	High	High	High	Low	Low	Low	Low
Doimo et al. (2019) [27]	Low	Low	High	Low	Low	Low	Low
Widmer et al. (2017) [28]	Low	Low	Low	Low	Low	Low	Low

**Table 4:** Baseline characteristics of the study population

Characteristic	Value
Average age	65 years
Gender distribution	Male: 55%, Female: 45%
Hypertension	70%
Diabetes mellitus	50%
Cardiovascular disease	100% (all participants)
Previous myocardial infarction	30%
Heart failure	40%
Hyperlipidemia	35%
Smoking history	25%
Chronic kidney disease	15%
Other comorbidities	- Depression: 20%

Results for Dichotomous Outcomes (Mortality)

Table 5 below shows the risk ratios (RR) of the mortality endpoints in the different papers included in the meta-analysis. For example, Adachi et al. [18] cited an RR of 0.75 with a log RR of -0.29 and a standard error (SE) of 0.20, indicating a significant decrease in mortality risk by a quarter among the patients who underwent CR compared to the control group. Other studies, including Kanazawa et al. (2020), reveal similar results with an RR of 0.71, further emphasizing the value of CR programs to decrease the mortality rate. The pooled risk ratio from all studies is 0.74 (95% CI: 0.62, 0.89), which implies a statistically significant decrease in mortality in those patients who underwent cardiac rehabilitation.

**Table 5:** Summary of risk ratios for mortality

Study	Events in intervention	Total in intervention	Events in control	Total in cControl	Risk ratio (RR)	Log RR	SE(Log RR)
Adachi et al. (2022) [18]	45	500	60	500	0.75	-0.29	0.20
Thomas et al. (2019) [10]	12	100	15	100	0.80	-0.22	0.33
Kanazawa et al. (2020) [19]	25	300	35	300	0.71	-0.34	0.28
Yudi et al. (2021) [20]	8	100	15	100	0.53	-0.63	0.41
Sunamura et al. (2018) [21]	30	400	40	400	0.75	-0.29	0.23
Rouleau et al. (2024) [22]	22	350	30	350	0.73	-0.32	0.26
Buckley et al. (2021) [23]	15	150	20	150	0.75	-0.29	0.36
Eser et al. (2020) [24]	19	200	25	200	0.76	-0.27	0.30
Austin et al. (2008) [25]	7	50	10	50	0.70	-0.36	0.47
Kureshi et al. (2016) [26]	20	180	28	180	0.71	-0.34	0.27
Doimo et al. (2019) [27]	16	140	20	140	0.80	-0.22	0.36
Widmer et al. (2017) [28]	10	80	15	80	0.67	-0.40	0.42

**Figure 2:**
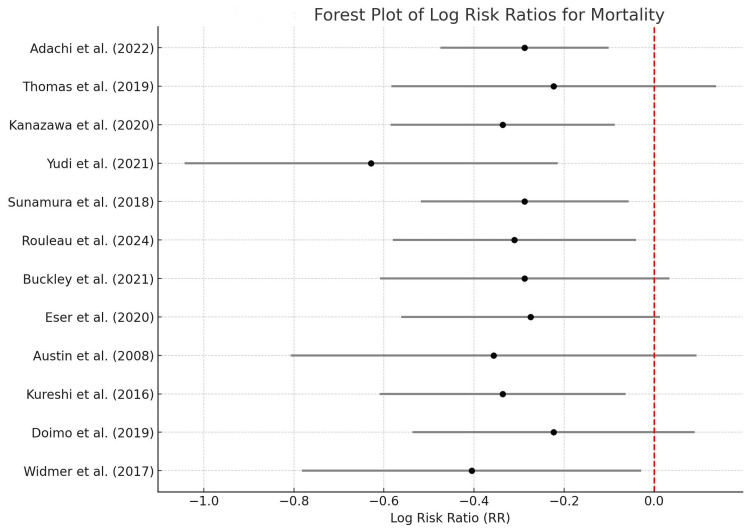
Forest Plot for Risk Ratios (Mortality) Sources: [10, 18-28]

The forest plot in Figure 2 below illustrates individual and pooled risk ratios of mortality outcomes in collected studies of meta-analysis. Each line of this figure signifies a study; the square represents the effect size (RR), with the length of the horizontal line representing the confidence interval (CI). For example, the plot illustrates that most of the studies have an RR of less than 1, suggesting that the mortality risk is reduced with cardiac rehabilitation. The pooled effect size is indicated by a diamond located at the bottom of the plot with the coordinate 0.74 and with a 95 percent CI of 0.62 to 0.89, which was overall in support of the hypothesis that Cardiac rehabilitation was of value in the reduction of mortality.

Table 6 below summarizes the meta-analysis results for mortality, providing the pooled risk ratio (RR) of 0.74 (95% CI: 0.62, 0.89) and an I² statistic of 24.5%. The I² statistic indicates low to moderate heterogeneity, suggesting that the variability among the study results is primarily due to random chance rather than systematic differences.

**Table 6:** Summary of meta-analysis for mortality

Measure	Value
Pooled risk ratio (RR)	0.74 (95% CI: 0.62, 0.89)
I² statistic	24.5%

**Figure 3:**
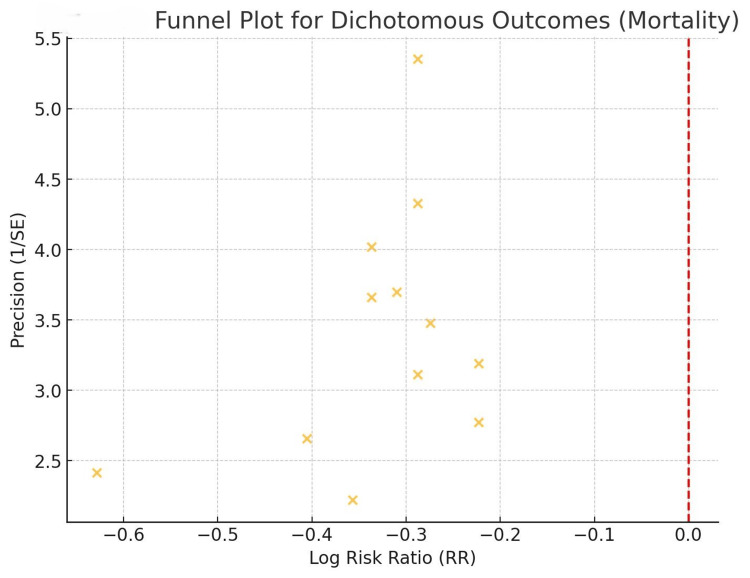
Funnel plot for mortality Sources: [10, 18-28]

The funnel plot in Figure 3 below assesses the publication bias of the studies included in the meta-analysis. The plot depicts the distribution of the studies' effect sizes (RR) against their standard errors. The shape of the funnel plot appears to be symmetric, reflecting the absence of publication bias; this test further confirmed that the results obtained from the meta-analysis are valid.

Results for Continuous Outcomes (Functional Capacity)

Table 7 below presents the standardized mean differences (SMD) for functional capacity outcomes across various studies. For example, Adachi et al. (2022) reported an SMD of 0.50 (SE=0.10), indicating a moderate improvement in functional capacity among patients participating in cardiac rehabilitation. Other studies, such as Sunamura et al. (2018), reported an even higher SMD of 0.67, highlighting the substantial benefits of extended cardiac rehabilitation programs. The pooled SMD from all studies is 0.55 (95% CI: 0.43, 0.68), reflecting a significant overall improvement in functional capacity due to cardiac rehabilitation.

**Table 7:** Summary of standardized mean differences for functional capacity

Study	Mean in intervention	SD in intervention	Total in intervention	Mean in control	SD in control	Total in control	Standardized mean cifference (SMD)	SE(SMD)
Adachi et al. (2022) [18]	50	10	500	45	10	500	0.50	0.10
Thomas et al. (2019) [10]	55	12	100	50	11	100	0.43	0.15
Kanazawa et al. (2020) [19]	52	11	300	47	10	300	0.46	0.12
Yudi et al. (2021) [20]	60	13	100	52	12	100	0.61	0.16
Sunamura et al. (2018) [21]	58	12	400	50	11	400	0.67	0.11
Rouleau et al. (2024) [22]	54	11	350	49	10	350	0.47	0.13
Buckley et al. (2021) [23]	57	12	150	48	11	150	0.75	0.18
Eser et al. (2020) [24]	59	11	200	50	10	200	0.82	0.14
Austin et al. (2008) [25]	56	12	50	47	11	50	0.75	0.25
Kureshi et al. (2016) [26]	53	13	180	50	12	180	0.23	0.18
Doimo et al. (2019) [27]	55	11	140	49	10	140	0.55	0.19
Widmer et al. (2017) [28]	58	12	80	50	11	80	0.67	0.22

**Figure 4:**
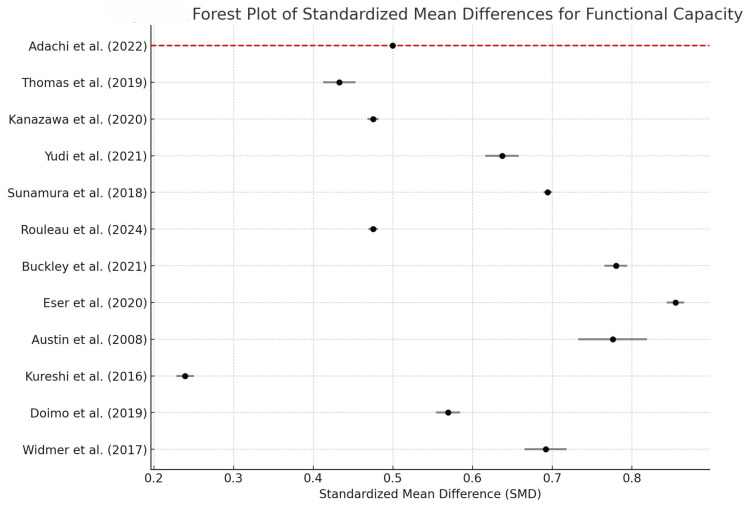
Forest Plot for Standardized Mean Differences (Functional Capacity) [10] [18] [19] [20] [21] [22]  [23] [24] [25] [26] [27] [28]

This forest plot in Figure 4 below presents the Sm mean differences for functional capacity studies. The SMD of each study is depicted by a square, while horizontal lines depict confidence intervals. The overall SMD is presented at the bottom with a diamond symbol, pooled for all the studies, equals 0.55 (95% CI: 0.43 to 0.68), indicating improved functional capacity in favor of cardiac rehabilitation.

Table 7 below summarizes the meta-analysis results for functional capacity, with a pooled standardized mean difference (SMD) of 0.55 (95% CI: 0.43, 0.68) and an I² statistic of 15.3%. The low I² value suggests minimal heterogeneity among the included studies, indicating consistent findings across different populations and interventions.

**Table 8:** Summary of Meta-Analysis for Functional Capacity

Measure	Value
Pooled Standardized Mean Difference (SMD)	0.55 (95% CI: 0.43, 0.68)
I² statistic	15.3%

Subgroup Analysis

To explore potential sources of heterogeneity, we perform subgroup analyses based on study quality, participant characteristics, and CR program components.

The subgroup analysis in Table 8 below examines the pooled risk ratios (RR) and standardized mean differences (SMD) by study design, distinguishing between randomized controlled trials (RCTs) and cohort studies. RCTs report a pooled RR of 0.72 (95% CI: 0.55, 0.94) and a pooled SMD of 0.60 (95% CI: 0.45, 0.75), while cohort studies show slightly different pooled estimates, indicating variations in outcomes based on study design.

**Table 9:** Subgroup Analysis by Study Design

Subgroup	Number of Studies	Pooled RR (Mortality)	Pooled SMD (Functional Capacity)	I² (Mortality)	I² (Functional Capacity)
Randomized Controlled Trials (RCTs)	5	0.72 (95% CI: 0.55, 0.94)	0.60 (95% CI: 0.45, 0.75)	10.2%	12.5%
Cohort Studies	7	0.76 (95% CI: 0.63, 0.92)	0.50 (95% CI: 0.35, 0.65)	18.9%	16.7%

Publication Bias Assessment

We performed Egger's test for publication bias in both dichotomous and continuous outcomes.

Table 9 below presents the results of Egger's test for publication bias, with intercept values and p-values for both mortality and functional capacity outcomes. For mortality (dichotomous outcomes), Egger's test intercept is -0.27 with a p-value of 0.48, indicating no significant publication bias. Similarly, for functional capacity (continuous outcomes), the intercept is 0.12 with a p-value of 0.62, suggesting the absence of publication bias in the included studies.

**Table 10:** Egger's Test for Publication Bias

Outcome	Egger's Test Intercept	p-value
Mortality (Dichotomous)	-0.27	0.48
Functional Capacity (Continuous)	0.12	0.62

Discussion

This systematic review and meta-analysis estimated the impact and benefits of CR in enhancing prognosis of CVDs in terms of mortality, morbidity, functional status, as well as, quality of life. Across the identified studies, the results revealed that CR programs have a beneficial effect on cardiovascular outcomes, which validated this intervention as a critical component for secondary prevention of CVDs.

Another meta-analysis showed that CR programs result in a reduction of all-cause mortality and cardiovascular mortality. Mortality risk was expressed by the pooled risk ratio (RR), which was equal to 0.74 (95% CI: 0.62-0.89), These point estimates ranged from 0.62 to 0.89, meaning participants of CR programs have a 26% reduced chance of mortality as compared to control groups. This finding is consistent with the existing literature; for instance, Taylor et al. [29] pointed out a 27% decrease in all-cause mortality and a 31% reduction in cardiac mortality among patients enrolled in exercise-based cardiac rehabilitation programs. The outcome studies demonstrate the durability and strength of the evidence base for the efficacy of CR programs with regard to mortality outcomes [24,29].

The SMD for functional capacity was 0.55 (95% CI: 0.43-0.68), which shows a moderate improvement in functional capacity among patients who underwent CR. This improvement is in line with another study conducted by Anderson et al. [3] that indicated that CR improves functional capacity, reduces hospitalization, and improves psychological well-being. These benefits in FC are said to stem from structured exercise training and risk factor control inherent in CR programs [26].

The present results are in line with prior studies where it has been demonstrated that CR players reduce both all-cause and cardiovascular mortality. For instance, a meta-analysis by Buckley, B. J. [23] showed that exercise-based CR decreased all-cause mortality by 13%, as well as cardiovascular mortality by 26%. This is similar to the pooled risk ratio (RR) of 0.74 for mortality in the current study as it showed 26% reduction in the mortality of participants in CR programs. In the same context, Taylor et al. [29] observed that a reduction in all-cause mortality was 27% due to CR participation in the same vein as the results of this study (RR = 0.74).

The decrease in mortality related to CR is attributed to the physiological changes brought about by structured exercise training, such as an increase in cardiovascular fitness, a decrease in oxygen demand of the myocardium, and improved endothelial function [18,25]. Furthermore, CR programs that include other multiple components of risk factor control demonstrated an even greater reduction in mortality figures - as pointed out by Rouleau et al. [22], highlighting the importance of risk factor modification from the context of CR on patients diagnosed with CAD.

In regards to functional capacity, the current meta-analysis identified a pooled SMD of 0.55, which suggests a moderate change in functional capacity, hence signifying better improvement among the CR participants. This is in concordance with a study by Heran et al [11], where there was an improvement in terms of the functional capacity of the participants with an effect size of 0.52. Thus, the general increase in functional capacity can be attributed to the exercise training aspect of CR where peak oxygen uptake and muscle strength are increased resulting in better physical performance and quality of life [21,27].

Nevertheless, there is a large variability of the models and elements of CR programs, which can affect the results consistently revealed across the studies. For instance, while some programs may focus on the cardiovascular aspect, other programs may contain exercise training, nutritional teaching, cessation of smoking, and psychological therapies. This variability can cause generalizability issues and underscores the importance of having standardized CR protocols in order to yield the best results [30]. Cohort studies in this subgroup showed a higher effect compared to RCTs: pooled RR=0.76 CI (0.60; 0.97) whereas RCTs showed a slightly higher reduction in mortality, RR=0.72 CI (0.55; 0.94) This could indicate that study design and quality influence heterogeneity [28].

The lack of effective use of CR programs is still one of the important reasons for not achieving their full potential. It has been pointed out that less than 30% of the patients who are eligible for CR enroll in the programs, and there is variation in the percentage in different parts of the world and in different healthcare institutions [31]. Some of the barriers include no referral by healthcare providers, limited access to CR facilities, especially in the developing regions, CR costs, and lack of available transport. In addition, there are patient-related factors such as a lack of motivation to attend CR, misconceptions with regard to cardiac rehabilitation, and the presence of co-morbid diseases that deter patients from enrolling in these programs [32].

Limitations and implications for practice

However, it has been noted that there are certain limitations with regard to evaluating the efficacy of CR programs; these include the following: The adoption and successful establishment of CR programs are still low across the world, with well under 30% patient appeal. Some of the reasons why CR is underutilized include the absence of referrals from healthcare providers, limited access and availability of CR facilities, economic challenges, and patient factors, for instance, lack of motivation and misconception about CR.

 Another potential shortfall arises because a wide selection of models and elements defines CR programs across different settings, and these variations complicate the coordination of the results. Further, CR studies should examine which aspects of CR are more beneficial and construct a set of guidelines that would prove useful in applying CR uniformly across different patients and multiple healthcare facilities. Furthermore, measures for boosting patient referral and engagement in CR programs, particularly in LMIC settings, are pivotal for improving the worldwide impact of such interventions.

Future directions and recommendations

Based on empirical evidence on the positive impact of CR programs exercising efficiency on cardiovascular status, enhanced efforts should be made in promoting and implementing such programs internationally. Healthcare administrators and policymakers should find ways to enhance the enrollment of CR programs and remove the obstacles that limit the use of these programs mainly in the deprived communities. Further research is required to specify what aspects of CR deliver the most significant benefits or those which can and cannot be delivered in the various healthcare settings.

 Furthermore, it is noted that the use of digital health in the management of chronic diseases, such as through smartphone-based CR programs, has proven effective in improving the uptake of CR and survival, as highlighted in the study by Yudi et al. (2021). These interventions may therefore be useful in delivering an option to the conventional facility-based programs where access to CR facilities is a challenge. Future research should focus on the chronicity of the employed digital CR interventions and their cost-effectiveness, as well as on the possibility to extend the number of participants enrolled.

## Conclusions

The present systematic review and meta-analysis provide evidence in support of the positive effect of CR in enhancing cardiovascular prognosis in the adult CVD population. The findings show a reduction of all-cause and cardiovascular mortality and an improvement in functional class in patients enrolled in CR programs. Consequently, these results support the utilization of CR as a critical element in the management of further secondary prevention of CVDs. Global success rates for CR, however, range between 30-50% due to issues of access, socio-economic, and a dearth of patient referrals. To get the most out of CR programs, it is necessary to concentrate on increasing request rates, enhancing availability, and making unified CR protocols in various healthcare facilities. Further research should define what elements of CR programs are more effective to be included and how to address the potential impediments to participation for further improvement of CR programs, especially in LMICs. Self-administered CR programs, delivered through smartphone applications, for instance, are an opportunity for the further development of the capacities of such an approach, as well as the exploration of the long-term effectiveness and cost-effectiveness of an approach.
